# Food Microbiology 2020

**DOI:** 10.1155/2021/9785432

**Published:** 2021-12-17

**Authors:** Marta Laranjo, María de Guía Córdoba, Teresa Semedo-Lemsaddek, Maria Eduarda Potes

**Affiliations:** ^1^MED-Mediterranean Institute for Agriculture, Environment and Development, Universidade de Évora, Évora, Portugal; ^2^Instituto Universitario de Investigación en Recursos Agrarios (INURA), Escuela de Ingenierías Agrarias, Universidad de Extremadura, Avd. Adolfo Suarez s/n, 06007 Badajoz, Spain; ^3^CIISA – Centro de Investigação Interdisciplinar em Sanidade Animal, Faculdade de Medicina Veterinária, Universidade de Lisboa, Avenida da Universidade Técnica, 1300-477 Lisboa, Portugal; ^4^Departamento de Medicina Veterinária, Escola de Ciências e Tecnologia, Universidade de Évora, Évora, Portugal

Food microbiology studies the microbiota of food products. It is a very diverse area within the field of microbiology because it deals with microorganisms that may have both beneficial and deleterious effects on food quality and safety, comprising fermentative, probiotic, spoilage, and pathogenic bacteria, moulds, yeasts, and other microorganisms.

The survival and growth of these diverse microorganisms is very much influenced by a wide range of environmental factors, which include the hugely diverse composition of the different food matrices.

Among the submitted manuscripts that pretty much covered all aspects of food microbiology, eight research articles were selected by external experts to enter this annual special issue of BioMed Research International.

Salameh et al. did a comparative study on the volatile oils of *Micromeria fruticosa serpyllifolia* plants, growing in different regions of Palestine, screening for their antioxidant and antimicrobial activities.

Gebru and Sbhatu studied a collection of probiotic lactic acid bacteria (LAB) isolated from Korean Kimchi and Ethiopian spontaneously fermented Teff and evaluated the effect of LAB on the phenolic content of Teff during fermentation.

The work by Su and coworkers evaluated the water-soluble polysaccharides of *Cordyceps kyushuensis*, a parasitic fungus, for their immunomodulatory and antioxidant actions and concluded that these could be considered potential candidates for functional foods and therapeutic agents.

Hassan and colleagues studied the probiotic properties of lactobacilli isolated from traditional Pakistani yoghurt and evaluated the antimicrobial activity of their bacteriocins against foodborne pathogens, such as *Staphylococcus aureus* and *Acinetobacter baumannii*.

Jaja et al. reported a high-level contamination of meat with *Staphylococcus aureus* and *Escherichia coli* resistant isolates, highlighting the public health consequences associated with the consumption of these unhygienic food products.

The article by Tenea described the role of peptide extracts from native LAB as promising food antimicrobials against *Salmonella enterica*.

Finally, Luo and colleagues described the beneficial effect of *Bacillus megaterium*-coated diets on the growth, digestive enzyme activity, and gut microbial diversity of Songpu mirror carp.

The authors come from nine different countries, one European (United Kingdom) and eight non-European, namely, Ethiopia, Pakistan, Saudi Arabia, South Africa, Nigeria, China, State of Palestine, and Equator.

We are happy to launch this special issue, which includes eight manuscripts that reported new findings mainly on the antimicrobial and antioxidant activities of food microbiota or their secondary metabolites. We hope that the readers of *BioMed Research International* find this Food Microbiology 2020 special issue of relevance to their research field.

